# Paclitaxel-Coated Balloon for Coronary In-Stent Restenosis and De Novo Disease in Chinese Patients

**DOI:** 10.1016/j.jacasi.2026.06.008

**Published:** 2026-08-01

**Authors:** Tse-Min Lu, Hsu-Lung Jen, Hee Hwa Ho, Chiung Ray Lu, Cheng-Ting Tsai, Feng-Yu Kuo, Simon Cheung-Chi Lam, Ji-Hung Wang, Jack Livingston, Rafael Cavalcante, Hsien-Li Kao

**Affiliations:** a Taipei Veterans General Hospital, Taipei, Taiwan; b Cheng Hsin General Hospital, Taipei, Taiwan; c Tan Tock Seng Hospital, Singapore; d China Medical University Hospital, Taichung, Taiwan; e Mackay Memorial Hospital, Taipei, Taiwan; f Kaohsiung Veterans General Hospital, Kaohsiung, Taiwan; g Department of Medicine, Queen Mary Hospital, Hong Kong SAR, China; h Hualien Tzu Chi Hospital, Hualien, Taiwan; i Boston Scientific Corporation, Marlborough, Massachusetts, USA; j National Taiwan University Hospital, Taipei, Taiwan

**Keywords:** Chinese population, de novo coronary disease, drug-coated balloon, in-stent restenosis, paclitaxel-coated balloon, percutaneous coronary intervention

## Abstract

**Background:**

Drug-coated balloons provide antiproliferative therapy without permanent implants and may benefit lesion subsets where drug-eluting stent performance is suboptimal.

**Objectives:**

The authors aimed to evaluate safety and effectiveness of a paclitaxel-coated balloon in a real-world cohort of ethnically Chinese patients with in-stent restenosis (ISR) or de novo coronary artery disease.

**Methods:**

This was a prospective, multicenter, single-arm registry across 9 sites in Taiwan, Hong Kong, and Singapore. Patients undergoing percutaneous coronary intervention with paclitaxel-coated balloon (AGENT, Boston Scientific Corp.) for ISR or de novo coronary artery disease were included. Primary endpoint was 12-month major adverse cardiac events (MACEs; cardiac death, myocardial infarction, or target vessel revascularization). Secondary endpoints included target lesion revascularization, target lesion thrombosis, and 9-month angiographic outcomes. Events adjudicated by independent clinical events committee; angiography analyzed by independent core laboratory.

**Results:**

A total of 500 patients (mean age 65.6 ± 11.2) were enrolled. At 365 days (IQR: 354-379) after the index procedure, 13.9% (68 of 489) of patients experienced MACE. MACE rates were 16.3% (48 of 294) and 10.5% (20 of 191), in ISR and de novo patients, respectively. Cardiac death and myocardial infarction rates were 1.6% (8 of 489) and 2.0% (10 of 489), respectively; target vessel revascularization occurred in 11.7% (57 of 489). Angiographic follow-up was performed in 35% (175 of 500) of patients. The 12-month MACE was significantly higher in patients undergoing surveillance angiography compared to without (26.9% [47 of 175] vs 4.9% [15 of 305], *P* < 0.001), driven by increased target lesion revascularization.

**Conclusions:**

Paclitaxel-coated balloon was safe and effective in ethnically Chinese patients. Surveillance imaging significantly influenced revascularization rates, underscoring its impact on clinical decision-making. (An "All-Comers" Study of the AgentTM MONORAILTM Paclitaxel-Coated PTCA Balloon Catheter Used in Real-World Clinical Practice in Chinese Patients; NCT04085445)

New-generation drug-eluting stents (DES) are the current standard for the percutaneous treatment of coronary artery disease. However, despite early efficacy, the implant of a permanent metallic scaffold is associated with a persistent risk of late failure of approximately 1% to 2% per year.^1^ These concerns are magnified for DES in certain clinical scenarios such as in-stent restenosis (ISR), particularly of multiple layers, as well as de novo small vessel disease, bifurcated lesions, and long lesion segments where complexity is heightened and the risk of recurrent restenosis is increased.

Drug-coated balloons (DCBs) have emerged as a compelling alternative to DES in selected settings. By delivering antiproliferative therapy without leaving behind a permanent implant, DCBs offer a "leave nothing behind" strategy that is particularly attractive in patients at high risk of restenosis or in whom further stenting is undesirable.^2^, ^3^, ^4^, ^5^, ^6^ The concept has been clinically validated in ISR, notably through AGENT IDE (A Clinical Trial to Assess the Agent Paclitaxel Coated PTCA [Percutaneous Transluminal Coronary Angioplasty] Balloon Catheter for the Treatment of Subjects With In-Stent Restenosis), which demonstrated the superiority of paclitaxel-coated balloon over uncoated balloon in patients with successful predilation.^2^

In de novo lesions, evidence is more limited. Although a comprehensive meta-analysis of randomized trials has supported the noninferiority of paclitaxel-coated balloons vs DES in selected small vessel disease populations, not all platforms have demonstrated consistent results.^4^ For example, recent trials such as REC-CAGEFREE I (Paclitaxel-Coated Balloon for Treatment of De-Novo Noncomplex Coronary Artery Lesions) failed to confirm noninferiority of another paclitaxel-coated balloon to DES in Chinese patients, highlighting the lack of a class effect among DCB technologies.^7^ Consequently, data specific to each DCB platform are needed, particularly in diverse patient populations and across various lesion subsets.

The paclitaxel-coated balloon (AGENT™, Boston Scientific Corp) is the first DCB approved by the Food and Drug Administration and has been in clinical use in Europe since 2014, demonstrating favorable outcomes across a range of lesion subsets.^8^^,^^9^ However, clinical evidence in additional patient populations remains limited. The present study describes performance, safety, and effectiveness of the paclitaxel-coated balloon among ethnically Chinese patients.

## Methods

### Trial design

This was a prospective, open-label, single-arm study designed to assess the safety and effectiveness of percutaneous coronary intervention (PCI) with a paclitaxel-coated balloon (AGENT™, Boston Scientific Corp) in an all-comer cohort of ethnically Chinese patients. All enrolled patients had ischemic symptoms or evidence of myocardial ischemia, were clinically indicated for PCI, and were treated with the study device. Major exclusion criteria included pregnancy, known intolerance to paclitaxel, or contraindication for dual-antiplatelet therapy.

This study (NCT04085445) adhered to the ISO 14155 standard for clinical investigations of medical devices, relevant sections of the International Council for Harmonisation Guidelines for Good Clinical Practice, ethical principles with origins in the Declaration of Helsinki, Chinese Medical Device Good Clinical Practice and pertinent individual country laws and regulations and directives at the time. The study was conducted after institutional review board approval at each site according to local and regional standard operating protocols. The principal investigators and co-authors have access to the data, drafted the initial version of the manuscript, and are responsible for the accuracy and completeness of the data and the integrity of this report.

### Clinical follow-up and outcomes

Scheduled clinical follow-up was planned at 30 days, 6 and 12 months, and then annually through 3 years post index procedure. Angiographic follow-up was planned at 9 months post index procedure for all patients with de novo lesions of reference vessel diameter ≤2.5 mm and for the first 100 consecutive patients with ISR lesions.

The primary endpoint was major adverse cardiac events (MACEs), defined as a composite of cardiac death, any myocardial infarction (MI) (Q-wave and non–Q-wave) and any target vessel revascularization (TVR) at 12 months post procedure. Additional prespecified secondary endpoints included the individual components of the primary endpoint, all-cause death, target lesion revascularization (TLR), and definite/probable target lesion thrombosis as defined by the Academic Research Consortium.^10^ An independent clinical events committee (Supplemental Table 1) adjudicated all clinical events included in the primary and key secondary endpoints. Repeat revascularization endpoints, including TLR and TVR, were not restricted to clinically driven events; therefore, revascularization could be performed following protocol-mandated surveillance angiography even in the absence of uniformly documented symptoms or objective ischemia.

Periprocedural endpoints included the rates of technical and procedural success. Baseline and 9-month surveillance angiograms were assessed by the independent core laboratories (Cardiovascular Imaging Core Laboratory, Boston, MA, USA, early in the trial with transition to BAIM Institute for Clinical Research, Inc, Boston MA, USA) and included in-stent (for the ISR group) and in-segment measurements of late loss, percent diameter stenosis, binary restenosis rate, and minimum lumen diameter (MLD).

### Statistical analysis

The present study was designed to describe clinical events and angiographic parameters in a meaningful patient population undergoing PCI with a paclitaxel-coated balloon. No formal sample size or power calculation was performed. Categorical variables are presented as counts with percentages, and continuous variables as mean ± SD and median with IQR, as appropriate. Binary incident outcomes, including technical success, procedural success, and MACEs, are reported as proportions with 2-sided 95% CIs. Time-to-event outcomes are reported as Kaplan-Meier estimates with corresponding 2-sided 95% CIs. Subgroup comparisons were assessed with chi-square tests or Fisher's exact tests, as appropriate and relative risk ratios (RRs) were calculated with the corresponding 95% CIs. CIs were not adjusted for multiplicity. Statistical analyses were performed using SAS software via SAS Enterprise Guide version 8.2.

## Results

Between November 2019 and August 2021, 500 all-comer patients were consecutively enrolled across 9 investigational sites in Taiwan, Hong Kong, and Singapore. Among them, 302 patients (357 lesions) were treated for ISR and 194 patients (227 lesions) were treated for de novo coronary artery disease. A total of 489 patients (97.8%) had clinical follow-up or death in the intention-to-treat analysis within 365 days (IQR: 354-379). One patient did not use the study device, 1 was lost to follow-up, 5 withdrew from the trial, 2 missed follow-up visits with no subsequent follow-up performed, and 2 missed the 1-year follow-up with later follow-up visits performed (Supplemental Figure 1).

Full baseline demographic and clinical characteristics are summarized in Table 1. The mean age of enrolled patients was 65.6 ± 11.2 years and 419 (83.8%) patients were male. Of the total patients, 451 (90.2%) were of Chinese ethnicity, whereas ethnicity was not disclosed for the remaining 49 (9.8%). Cardiovascular risk factors were prevalent, with diabetes present in 290 (58.0%) patients and hyperlipidemia in 403 (80.6%) patients. Coronary artery disease history was extensive, with 313 (62.6%) having a history of multivessel disease. The clinical presentation was chronic coronary syndrome in 283 (56.6%) patients. There were 16 patients with both ISR and de novo lesions, and they were included in the ISR group for the statistical analyses. Four patients were included in the overall cohort but were not included in the ISR or de novo groups (1 restenotic lesion without stent; 3 ST-segment elevation MI culprit lesions without documented lesion classification).

**Table 1:** Baseline Characteristics

	Overall N = 500	ISR n = 302	De Novo n = 194
Age, y	65.6 ± 11.2	66.8 ± 11.2	63.8 ± 11.2
Male	419 (83.8)	254 (84.1)	163 (84.0)
Chinese^a^	451 (90.2)	297 (98.3)	150 (77.3)
Current smoker	112 (22.4)	69 (22.8)	43 (22.2)
Previous smoker	163 (32.6)	112 (37.1)	51 (26.3)
Diabetes	290 (58.0)	192 (63.6)	98 (50.5)
Presentation			
CCS	283 (56.6)	156 (51.7)	125 (64.4)
ACS	213 (42.6)	143 (47.4)	68 (35.1)
Unknown	4 (0.8)	3 (1.0)	1 (0.5)
Silent ischemia	47 (9.4)	31 (10.3)	16 (8.2)
Medical history			
Medicated hyperlipidemia	403 (80.6)	254 (84.1)	147 (75.8)
Medicated hypertension	403 (80.6)	241 (79.8)	159 (82.0)
MI	174 (34.8)	124 (41.1)	50 (25.8)
CHF	119 (23.8)	84 (27.8)	34 (17.5)
PCI	411 (82.2)	299 (99.0)	110 (56.7)
CABG	21 (4.2)	19 (6.3)	1 (0.5)
Arrhythmia	59 (11.8)	34 (11.3)	25 (12.9)
Multivessel disease	313 (62.6)	222 (73.5)	89 (45.9)
Left main disease	79 (15.8)	60 (19.9)	19 (9.8)
Transient ischemic attacks	9 (1.8)	6 (2.0)	2 (1.0)
Cerebrovascular accidents	29 (5.8)	17 (5.6)	11 (5.7)
Renal disease	118 (23.6)	81 (26.8)	36 (18.6)
Peripheral vascular disease	37 (7.4)	27 (8.9)	10 (5.2)
Target vessel			
LAD	229 (45.8)	154 (51.0)	74 (38.1)
LCX	144 (28.8)	67 (22.2)	75 (38.7)
RCA	169 (33.8)	110 (36.4)	58 (29.9)
LM	11 (2.2)	10 (3.3)	1 (0.5)

Values are reported as n (%) or mean ± SD.

ACS = acute coronary syndrome; CABG = coronary artery bypass graft; CCS = chronic coronary syndrome; CHF = congestive heart failure; ISR = in-stent restenosis; LAD = left anterior descending; LCX = left circumflex; LM = left main; MI = myocardial infarction; PCI = percutaneous coronary intervention; RCA = right coronary artery.

a 100% patients who were asked self-reported Chinese ethnicity; unknown ethnicity in 49 patients.

### Baseline angiographic and procedural characteristics

The core laboratory reported quantitative coronary angiography (QCA) results at baseline, including in-segment and in-lesion measurements, summarized in Tables 2. At baseline, the mean reference vessel diameter was 3.0 ± 0.5 mm in the ISR group and 2.3 ± 0.3 mm in the de novo group (Table 2), 89.9% (204 of 227) of de novo lesions were of reference vessel diameter ≤2.5 mm. The mean lesion length was 23.4 ± 12.1 mm and 20.7 ± 10.1 mm for ISR and de novo groups, respectively. Device and procedural characteristics are summarized in Table 3, with postprocedure QCA results in Table 4. Technical success (defined by site-reported ability to cross and dilate the target lesion(s) with the study device and postprocedure residual angiographic stenosis ≤30%) was achieved in 98.7% (289 of 302, 95% CI: 96.6%-99.6%) of ISR patients and 86.1% (167 of 194, 95% CI: 80.4%-90.6%) of de novo patients. Procedural success (technical success with no death or MI within 24 hours) was achieved in 98.7% (298 of 302, 95% CI: 96.6%-99.6%) for ISR and 85.6% (166 of 194, 95% CI: 79.8%-90.2%) for de novo. The lower technical and procedural success rates in the de novo cohort were primarily driven by residual angiographic stenosis >30% rather than device delivery failure or early clinical events. Among the 27 de novo patients not meeting technical success, 24 had postprocedure diameter stenosis >30% and 3 were due to failure to cross/dilate the target lesion. Procedural success was not achieved in 28 de novo patients, reflecting the 27 technical failures plus 1 patient with death or MI within 24 hours. Bailout stenting was used in 7.6% (27 of 357) of ISR lesions and 7.0% (16 of 227) of de novo lesions.

**Table 2:** Preprocedure Lesion Characteristics

Characteristics^a^	Overall^b^ N = 500	ISR^c^ n = 302	De Novo n = 194
De novo lesions	243 (41.3)	18 (5.0)	225 (99.1)
Reference vessel diameter, mm	2.7 ± 0.5	3.0 ± 0.5	2.3 ± 0.3
Lesion length, mm	22.4 ± 11.5	23.4 ± 12.1	20.7 ± 10.1
% Diameter stenosis	83.0 ± 11.6	81.2 ± 12.1	85.7 ± 10.1
MLD, mm	0.8 ± 0.4 0.8 (0.6, 1.0)	0.9 ± 0.4 0.9 (0.7, 1.1)	0.7 ± 0.3 0.7 (0.5, 0.8)
TIMI flow			
0	71 (12.1)	52 (14.6)	18 (7.9)
1	91 (15.5)	67 (18.8)	23 (10.1)
2	159 (27.0)	80 (22.4)	79 (34.8)
3	266 (45.2)	157 (44.0)	107 (47.1)
Calcification			
Moderate	61 (10.4)	43 (12.0)	18 (7.9)
Severe	38 (6.5)	20 (5.6)	16 (7.0)
Tortuosity			
Moderate	42 (7.1)	15 (4.2)	27 (11.9)
Severe	9 (1.5)	0 (0.0)	9 (4.0)
Thrombus present	14 (2.4)	11 (3.1)	3 (1.3)

Values are reported as n (%), mean ± SD, or median (25th percentile, 75th percentile).

MLD = minimal lumen diameter; TIMI = thrombolysis in myocardial infarction. Other abbreviation as in Table 1.

a 588 lesions in overall patient cohort; 357 lesions in ISR group; 227 lesions in de novo group.

b 4 patients were included in the overall cohort but were not included in the ISR or de novo group (1 restenotic lesion no stent; 3 ST-segment elevation myocardial infarction culprit lesion).

c Patients with both ISR and de novo lesions were included in the ISR group.

**Table 3:** Device and Procedural Characteristics (Lesion-Level; Site Reported)

	Overall N = 500	ISR n = 302	De Novo n = 194
Device characteristics^a^			
Balloon diameter, mm	2.7 ± 0.6	3.0 ± 0.5	2.3 ± 0.4
Balloon to artery ratio	1.0 ± 0.1 1.0 (1.0, 1.0)	1.0 ± 0.1 1.0 (1.0, 1.0)	1.0 ± 0.1 1.0 (1.0, 1.0)
Balloon length, mm	24.1 ± 6.2	24.8 ± 5.9	23.1 ± 6.5
Device deployed	658 (99.4)	398 (100.0)	255 (98.5)
Max inflation pressure, atm	9.5 ± 3.3	10.0 ± 3.1	8.6 ± 3.4
Inflation time, s	61.3 ± 28.2	58.7 ± 27.0	65.4 ± 29.7
Device per lesion	1.1 ± 0.4 1.0 (1.0, 1.0)	1.1 ± 0.4 1.0 (1.0, 1.0)	1.1 ± 0.4 1.0 (1.0, 1.0)
Bailout stent used per lesion	43 (7.3)	27 (7.6)	16 (7.0)
Dissection	20 (3.0)	1 (0.3)	19 (7.3)
A	4 (0.6)	0 (0.0)	4 (1.5)
B	12 (1.8)	1 (0.3)	11 (4.2)
C	3 (0.5)	0 (0.0)	3 (1.2)
D	1 (0.2)	0 (0.0)	1 (0.4)
E/F	0 (0.0)	0 (0.0)	0 (0.0)
Technical success^b^	469 (93.8) [91.3-95.7]	298 (98.7) [96.6-99.6]	167 (86.1) [80.4-90.6]
Procedural success^c^	468 (93.6) [91.1-95.6]	298 (98.7) [96.6-99.6]	166 (85.6) [79.8-90.2]

Values are reported as n (%), mean ± SD, or median (25th percentile, 75th percentile); 95% confidence intervals for binary clinical outcomes were calculated using the Clopper-Pearson exact method, were not adjusted for multiplicity, and are reported as [lower confidence interval, upper confidence interval].

Abbreviation as in Table 1.

a 662 devices and 588 lesions for overall patient cohort; 398 devices and 357 lesions for ISR group; 259 devices and 227 lesions for de novo group.

b Technical success was defined as the ability to cross and dilate the lesion to achieve residual angiographic stenosis no greater than 30%.

c Procedural success was defined technical success with no death or myocardial infarction within 24 hours of the index procedure.

**Table 4:** Postprocedural Quantitative Coronary Angiography Measurements

Characteristics^a^	Overall N = 368	ISR n = 181	De Novo n = 184
Reference vessel diameter, mm	2.3 ± 0.5	2.5 ± 0.5	2.0 ± 0.4
MLD, mm			
In lesion	1.6 ± 0.5 1.54 (1.27, 1.88)	1.8 ± 0.4 1.79 (1.47, 2.11)	1.4 ± 0.4 1.29 (1.14,1.58)
In segment	1.6 ± 0.5 1.57 (1.28,1.94)	1.9 ± 0.4 1.81 (1.52, 2.20)	1.4 ± 0.4 1.32 (1.15, 1.61)
Acute gain, mm			
In lesion	0.9 ± 0.4 0.78 (0.56, 1.08)	1.0 ± 0.5 0.87 (0.68, 1.24)	0.7 ± 0.3 0.68 (0.52, 0.95)
In segment	0.9 ± 0.4 0.82 (0.60, 1.14)	1.0 ± 0.5 0.92 (0.70, 1.32)	0.8 ± 0.4 0.71 (0.53, 0.97)
Diameter stenosis, %			
In lesion	30.2 ± 11.3	28.7 ± 10.2	31.9 ± 12.2
In segment	28.3 ± 11.9	26.2 ± 10.6	30.5 ± 12.8
TIMI flow			
0/1	0 (0.0)	0 (0.0)	0 (0.0)
2	1 (0.2)	0 (0.0)	1 (0.5)
3	430 (98.4)	219 (98.6)	208 (98.1)
N/A	6 (1.4)	3 (1.4)	3 (1.4)

Values are reported as n (%), mean ± SD, or median (25th percentile, 75th percentile).

Abbreviations as in Tables 1 and 2.

a 437 lesions in overall patient cohort; 222 lesions in ISR group; 212 lesions in de novo group.

### Surveillance angiography outcomes

**Figure 1:**
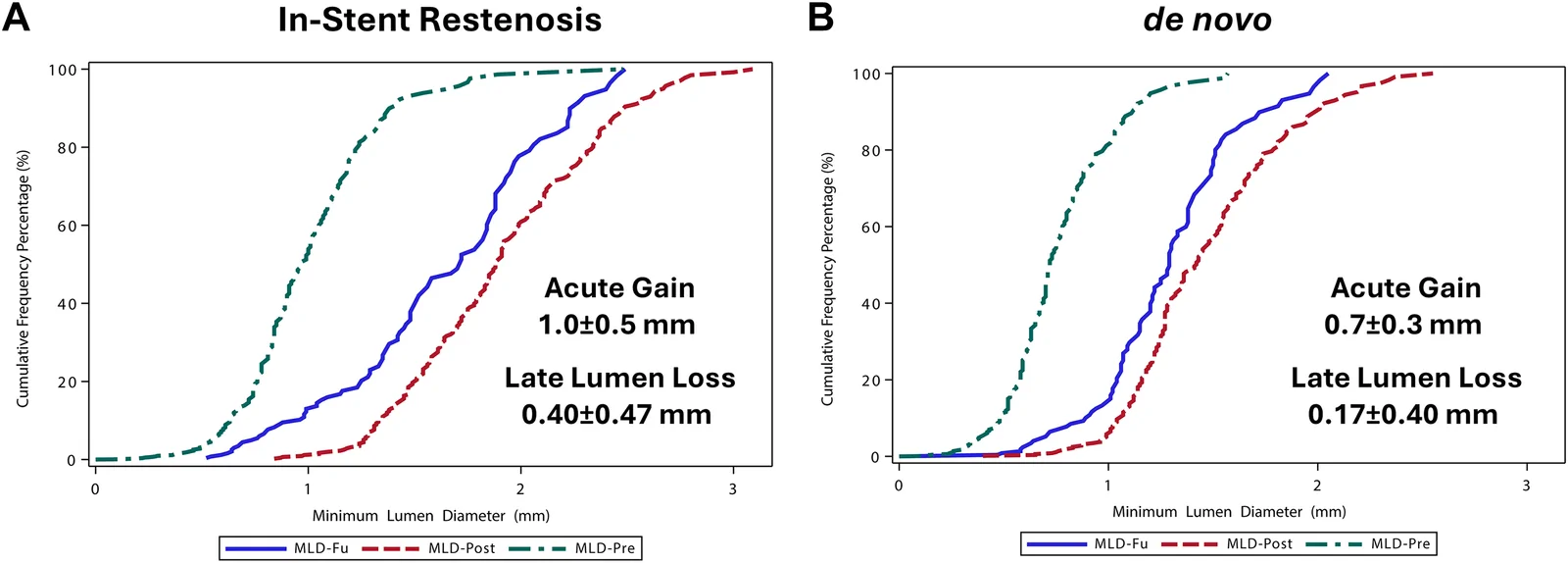
In-Lesion Minimum Lumen Diameters at Baseline, Postprocedure, and 9-Month Follow-Up (A) Patients with in-stent restenosis treated with paclitaxel-coated balloon at baseline, postprocedure, and 9-month follow-up. Acute gain in MLD from baseline to postprocedure was 1.0 ± 0.5 mm; 0.87 (0.68, 1.24). Late lumen loss from postprocedure to 9-month follow-up was 0.40 ± 0.47 mm; 0.35 (0.03, 0.71). (B) Patients with de novo lesions treated with paclitaxel-coated balloon at baseline, postprocedure, and 9-month follow-up. Acute gain in MLD from baseline to postprocedure was 0.7 ± 0.3 mm; 0.68 (0.52, 0.95). Late lumen loss in MLD from postprocedure to 9-month follow-up was 0.17 ± 0.40 mm; 0.07 (−0.10, 0.46). MLD = minimum lumen diameter

Detailed follow-up angiographic outcomes are presented in Table 5. At 9-month follow-up, core laboratory–assessed QCA was available for 34% (170 of 500) of patients. The rate of in-lesion binary restenosis was 38.1% (37 of 97) in ISR lesions and 21.8% (22 of 101) in de novo lesions. Mean in-lesion minimum lumen diameter was 1.5 ± 0.5 mm and 1.1 ± 0.4 mm for ISR and de novo groups, respectively. Corresponding medians were 1.48 (1.04-1.88) and 1.18 (0.97-1.38). Mean in-lesion late lumen loss was 0.4 ± 0.5 mm for ISR lesions and 0.2 ± 0.4 mm for de novo lesions. Corresponding medians were 0.35 (0.03-0.71) and 0.07 (−0.10 to 0.46) (Figure 1). Among 99 de novo lesions with 9-month late lumen loss measured, 37 (37.4%) experienced late lumen enlargement (LLE).

**Table 5:** 9-Month Follow-up Quantitative Coronary Angiography Measurements

Characteristics^a^	Overall N = 170	ISR n = 78	De Novo n = 90
Reference vessel diameter, mm	2.2 ± 0.5	2.6 ± 0.5	1.9 ± 0.3
MLD, mm			
In-lesion	1.3 ± 0.5 1.29 (0.99, 1.57)	1.5 ± 0.5 1.48 (1.04, 1.88)	1.1 ± 0.4 1.18 (0.97, 1.38)
In-segment	1.3 ± 0.5 1.33 (1.02, 1.69)	1.5 ± 0.5 1.48 (1.06, 1.88)	1.2 ± 0.5 1.20 (0.99,1.45)
Late lumen loss, mm			
In-lesion	0.28 ± 0.45 0.16 (−0.05,0.59)	0.40 ± 0.47 0.35 (0.03, 0.71)	0.17 ± 0.40 0.07 (−0.10, 0.46)
In-segment	0.29 ± 0.49 0.18 (−0.04, 0.60)	0.44 ± 0.51 0.32 (0.03, 0.83)	0.15 ± 0.43 0.07 (−0.17, 0.46)
Binary restenosis			
In-lesion	59 (29.5)	37 (38.1)	22 (21.8)
In-segment	58 (29.0)	36 (37.1)	22 (21.8)
% Diameter stenosis			
In-lesion	42.0 ± 18.6	43.1 ± 17.4	41.2 ± 19.8
In-segment	40.5 ± 19.8	41.9 ± 18.4	39.3 ± 21.3
TIMI flow			
0	6 (3.0)	1 (1.0)	5 (5.0)
1	0 (0.0)	0 (0.0)	0 (0.0)
2	1 (0.5)	1 (1.0)	0 (0.0)
3	193 (96.5)	95 (97.9)	96 (95.0)

Values are reported as n (%), mean ± SD, or median (25th percentile, 75th percentile).

Abbreviations as in Tables 1 and 2.

a 200 lesions in overall patient cohort; 97 lesions in ISR group; 101 lesions in de novo group.

### Primary endpoint and additional outcomes

**Figure 2:**
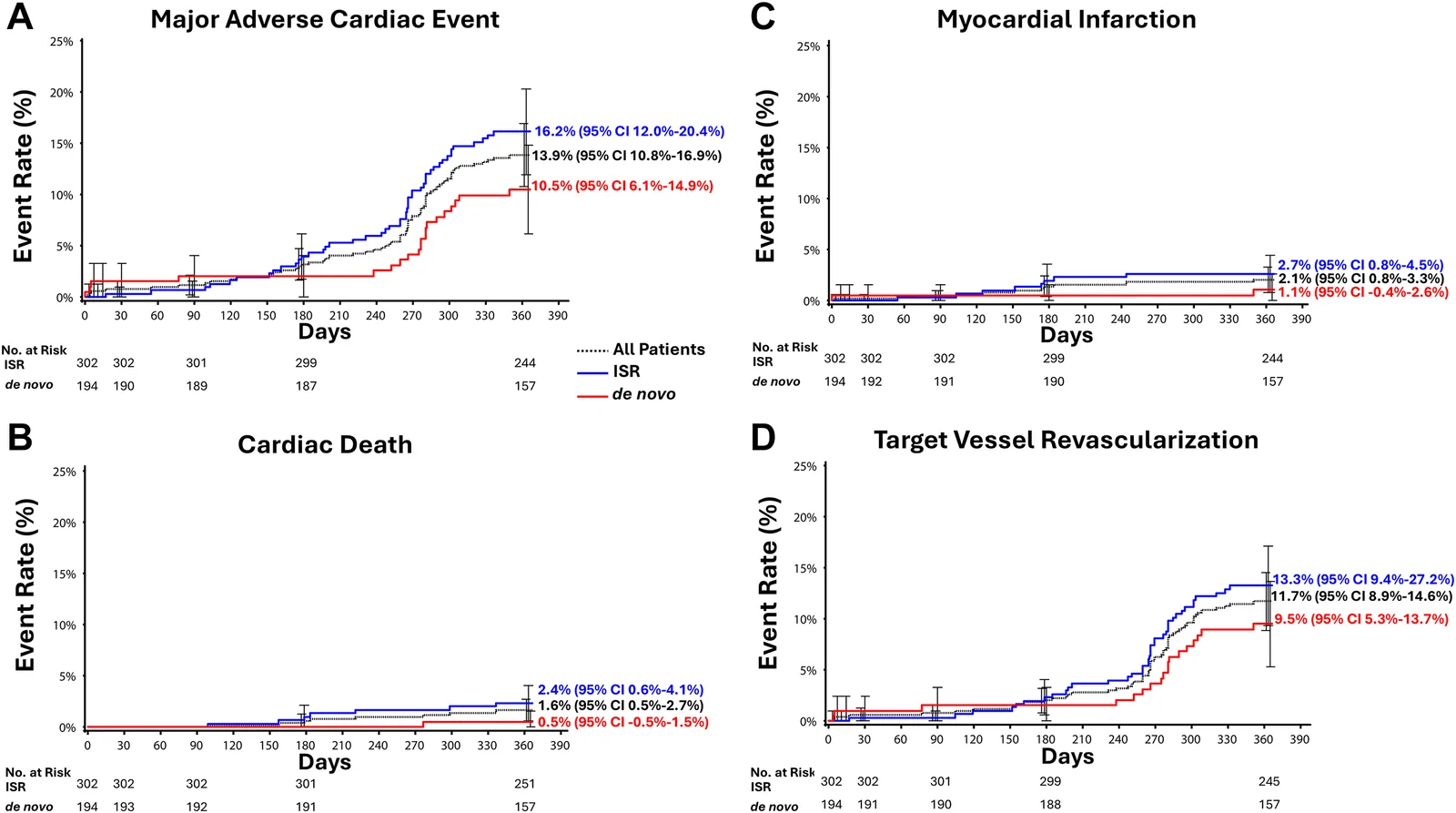
12-Month Major Adverse Cardiac Events and Component Endpoints (A) Kaplan-Meier major adverse cardiac event rates at 12 months were 13.9% (95% CI: 10.8%-16.9%) overall, 16.2% (95% CI: 12.0%-20.4%) for ISR, and 10.5% (95% CI: 6.1%-14.9%) for de novo. (B) Kaplan-Meier cardiac death rates at 12 months were 1.6% (95% CI: 0.5%-2.7%) overall, 2.4% (95% CI: 0.6%-4.1%) for ISR, and 0.5% (95% CI: −0.5% to 1.5%) for de novo. (C) Kaplan-Meier myocardial infarction rates at 12 months were 2.1% (95% CI: 0.8%-3.3%) overall, 2.7% (95% CI: 0.8%-4.5%) for ISR, and 1.1% (95% CI: −0.4% to 2.6%) for de novo. (D) Kaplan-Meier target vessel revascularization rates at 12 months were 11.7% (95% CI: 8.9%-14.6%) overall, 13.3% (95% CI: 9.4%-17.2%) for ISR, and 9.5% (95% CI: 5.3%-13.7%) for de novo. ISR = in-stent restenosis.

At the 1-year follow-up, the overall incidence of MACE was 13.9% (68 of 489, 95% CI: 11.0%-17.3%). The Kaplan-Meier estimate of cumulative MACE at 1 year is shown in Figure 2A. Stratified by lesion type, MACE occurred 16.3% (48 of 294, 95% CI: 12.3%-21.1%) of patients with ISR and 10.5% (20 of 191, 95% CI: 6.5%-15.7%) of patients with de novo lesions.

Cardiac death at 1 year occurred in 1.6% (8 of 489, 95% CI: 0.7%-3.2%) of the total patient population: 2.4% (7 of 294, 95% CI: 1.0%-4.8%) in the ISR group and 0.5% (1 of 191, 95% CI: 0.0%-2.9%) in the de novo group. The cumulative incidence of MI occurred in 2.0% (10 of 489, 95% CI: 1.0%-3.7%) of the total patient population with 2.7% (8 of 294, 95% CI: 1.2%-5.3%) of patients from the ISR group and 1.0% (2 of 191, 95% CI: 0.1%-3.7%) from the de novo group. TVR was observed in 11.7% (57 of 489, 95% CI: 8.9%-14.8%) of patients of the total cohort, comprising 13.3% (39 of 294, 95% CI: 9.6%-17.7%) of ISR and 9.4% (18 of 191, 95% CI: 5.7%-14.5%) of de novo patients. Additional 12-month clinical outcomes are summarized in Table 6.

**Table 6:** 12-Month Follow-Up Clinical Outcomes After Treatment With Paclitaxel-Coated Balloon

	Overall N = 489	ISR n = 294	De Novo n = 191
MACE	68 (13.9) [11.0-17.3]	48 (16.3) [12.3-21.2]	20 (10.5) [6.5-15.7]
Cardiac death	8 (1.6) [0.7-3.2]	7 (2.4) [1.0-4.8]	1 (0.5) [0.0-2.9]
Myocardial infarction	10 (2.0) [1.0-3.7]	8 (2.7) [1.2-5.3]	2 (1.0) [0.1-3.7]
Q-wave	1 (0.2) [0.0-1.1]	0 (0.0) [0.0-1.2]	1 (0.5) [0.0-2.9]
Non-Q-wave	9 (1.8) [0.8-3.5]	8 (2.7) [1.2-5.3]	1 (0.5) [0.0-2.9]
Target vessel revascularization	57 (11.7) [8.9-14.8]	39 (13.3) [9.6-17.7]	18 (9.4) [5.7-14.5]
Any target lesion revascularization	50 (10.2) [7.7-13.3]	34 (11.6) [8.1-15.8]	16 (8.4) [4.9-13.2]
Definite/Probable thrombosis	1 (0.2) [0.0-1.1]	1 (0.3) [0.0-1.9]	0 (0.0) [0.0-1.9]

Values are reported as n (%); 95% CIs for binary clinical outcomes were calculated using the Clopper-Pearson exact method, were not adjusted for multiplicity, and are reported as [lower confidence interval, upper confidence interval].

MACE = major adverse cardiac events; other abbreviation as in Table 1.

### Subgroup analyses

**Figure 3:**
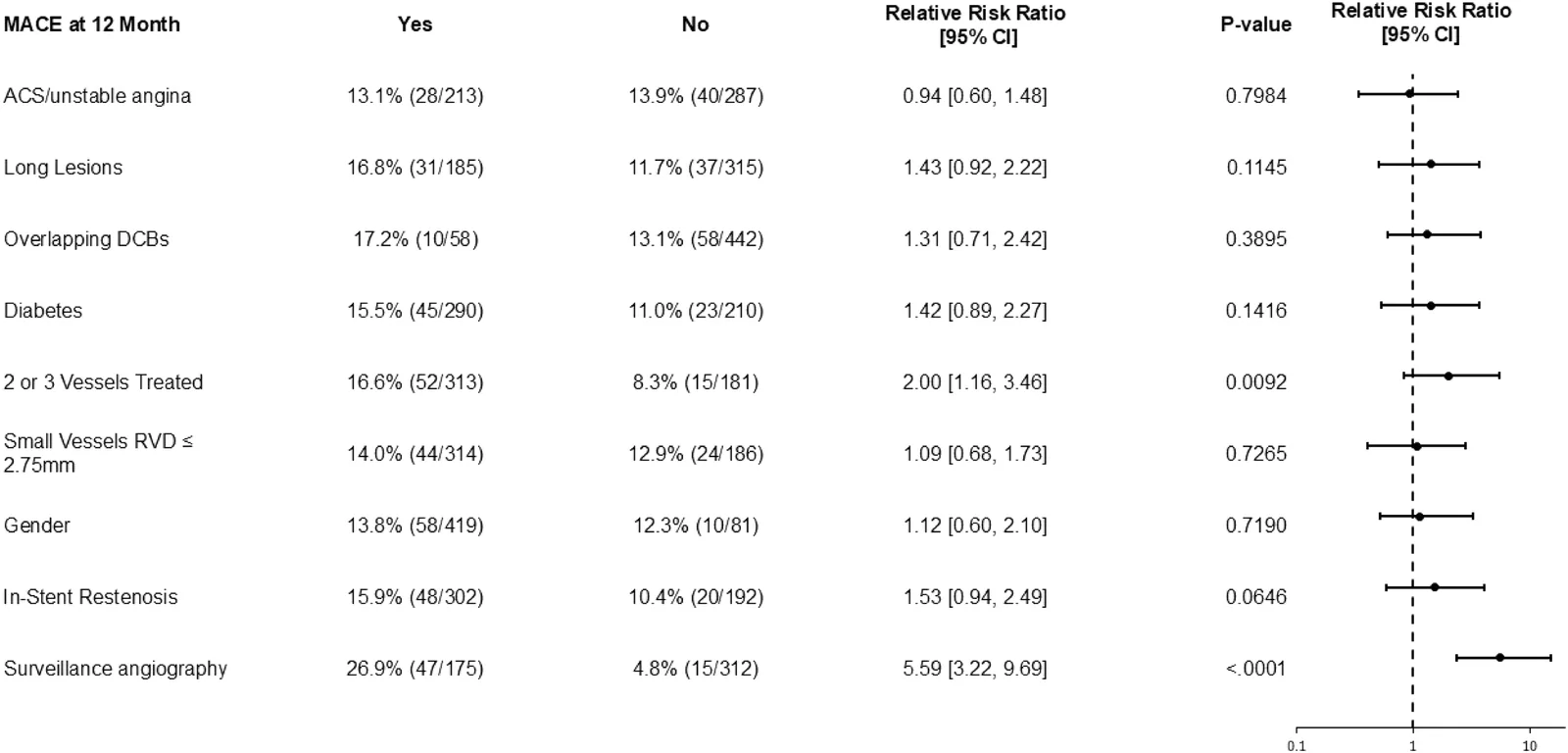
Forest Plot of 12-Month MACE for All Patients No category was designated as the reference group for the relative risk ratio calculations. Sex was coded as “Yes” for male and “No” for female in the analysis. The x-axis is log transformed. *P* value for the subgroup comparison calculated from a chi-square test. ACS = acute coronary syndrome; DCB = drug-coated balloon; MACE = major adverse cardiac event; RVD = reference vessel diameter

**Figure 4:**
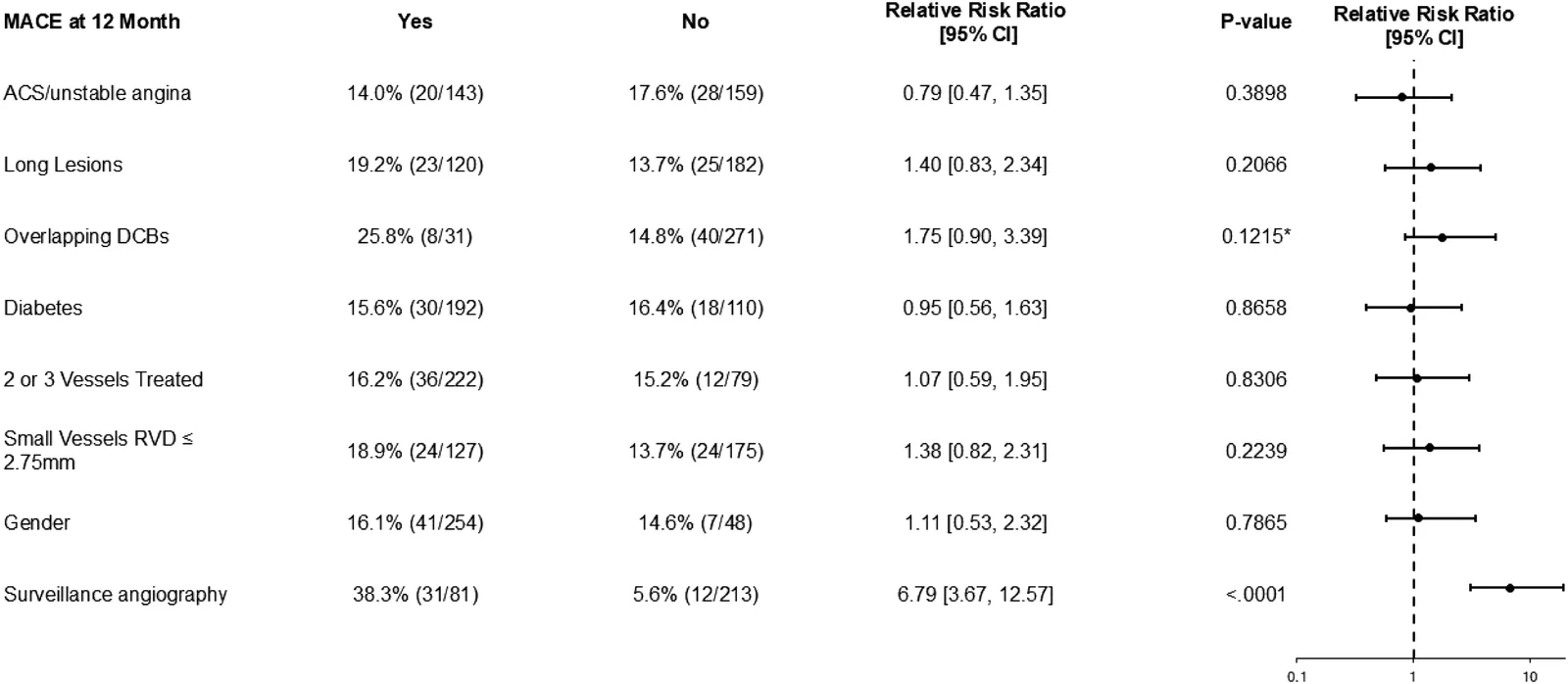
Forest Plot of 12-Month MACE for In-Stent Restenosis Patients No category was designated as the reference group for the relative risk ratio calculations. Sex was coded as “Yes” for male and “No” for female in the analysis. The x-axis is log transformed. *P* value for the subgroup comparison calculated from a chi-square test or ∗Fisher's exact test, as appropriate. Abbreviations as in Figure 3.

**Figure 5:**
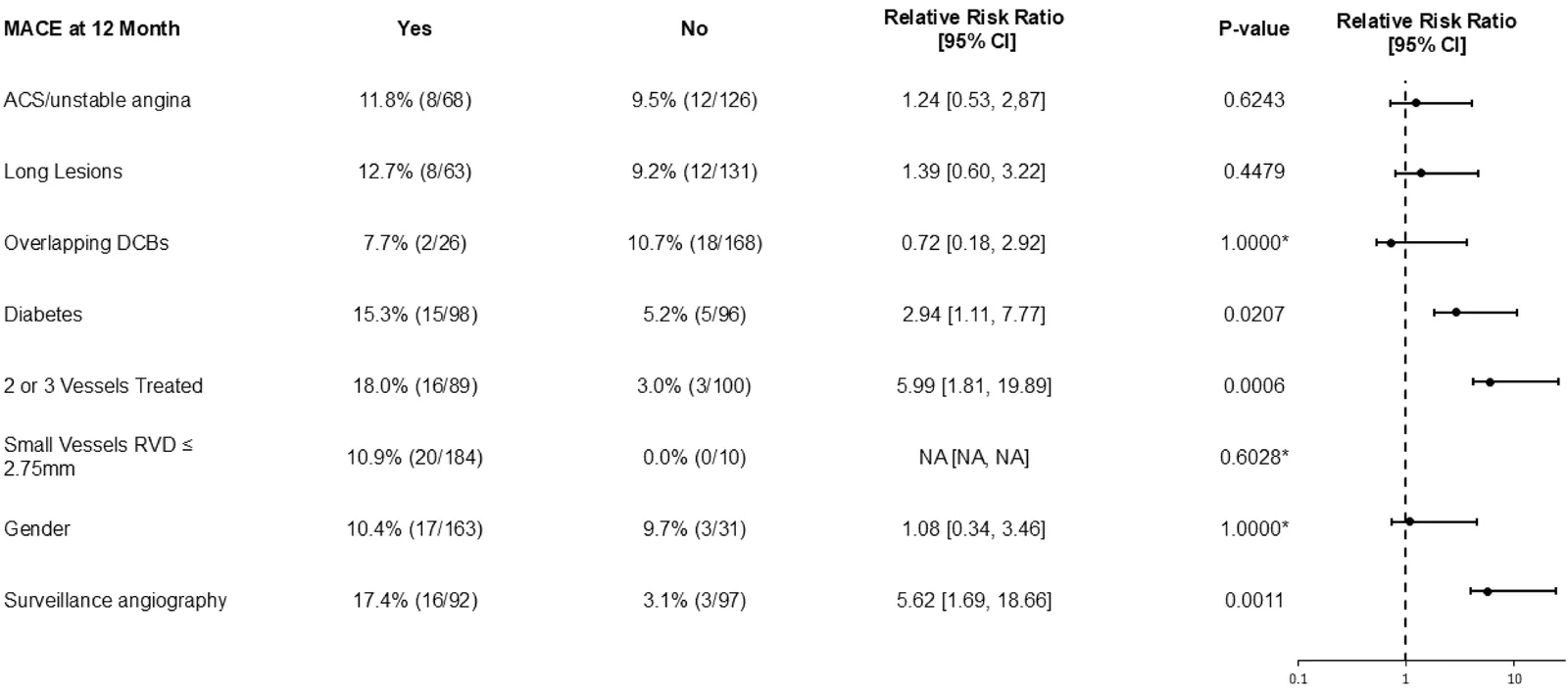
Forest Plot of 12-Month MACE for De Novo Patients No category was designated as the reference group for the relative risk ratio calculations. Sex was coded as “Yes” for male and “No” for female in the analysis. The x-axis is log transformed. *P* value for the subgroup comparison calculated from a chi-square test or ∗Fisher's exact test, as appropriate. Abbreviations as in Figure 3.

Subgroup analyses are presented in Figure 3, Figure 4, Figure 5. Clinical outcomes were generally consistent across multiple prespecified subgroups, including male vs female patients, ISR vs de novo lesions, as well as those with acute coronary syndrome and long lesions (>25 mm). There was a trend toward higher 12-month MACE in ISR vs de novo patients (RR: 1.53; 95% CI: 0.94-2.49). Within the de novo subgroup, higher 12-month MACE risk was observed in patients with diabetes vs those without diabetes (RR: 2.94; 95% CI: 1.11-7.77) and in patients with 2- or 3-vessel treatment vs single-vessel treatment (RR: 5.99; 95% CI: 1.81-19.89).

### Clinical impact of surveillance angiography

**Figure 6:**
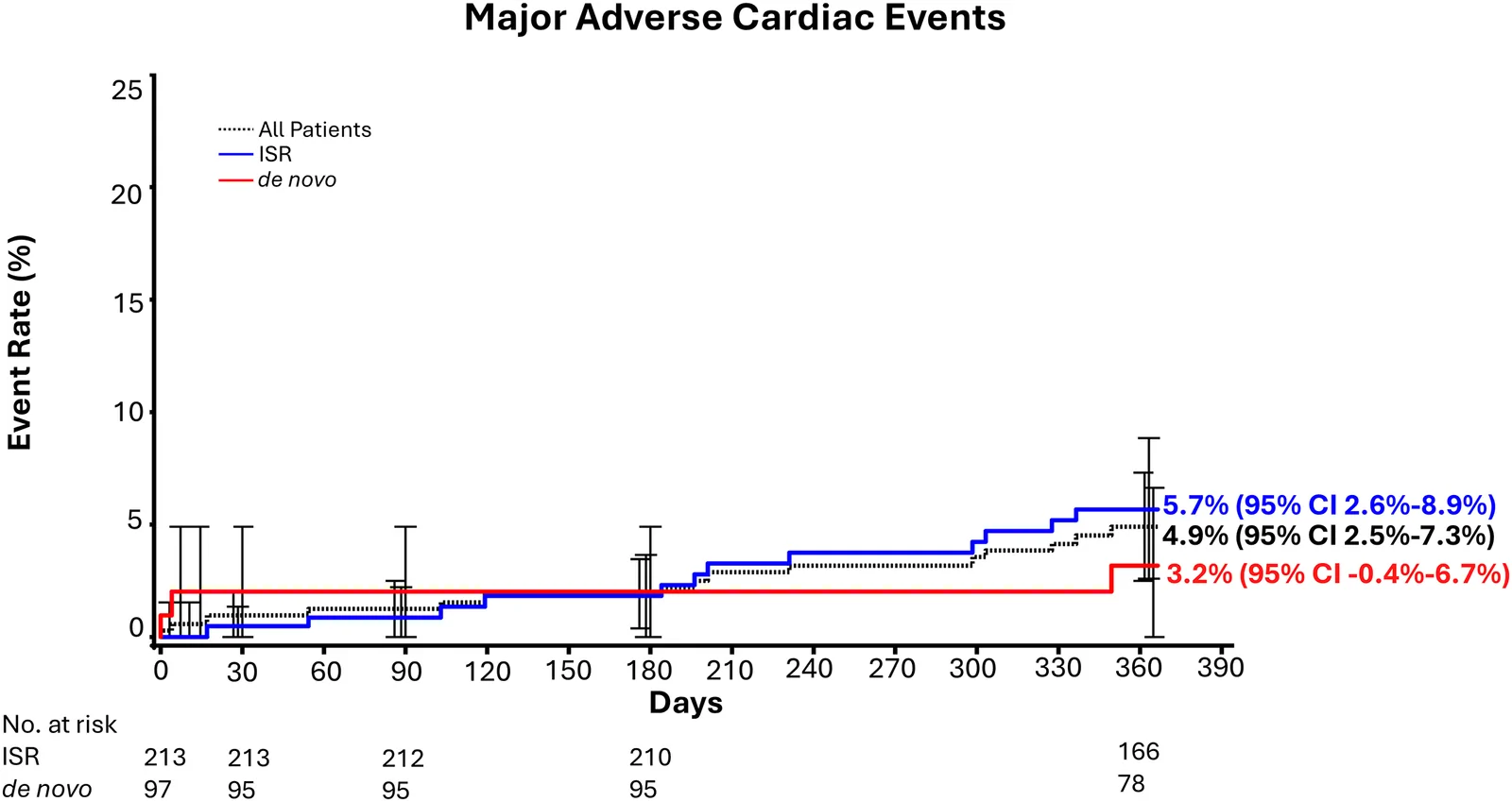
12-Month Major Adverse Cardiovascular Events for Patients Without Surveillance Angiography Major adverse cardiac event was defined as the composite of cardiac death, myocardial infarction, and target vessel revascularization. Kaplan-Meier event rates at 12 months were 4.9% (95% CI: 2.5%-7.3%) overall, 5.7% (95% CI: 2.6%-8.9%) ISR, and 3.2% (95% CI: 0%-6.7%) de novo. Abbreviation as in Figure 2.

The incidence of MACE in patients who underwent scheduled surveillance angiography was 26.9% (47 of 175, 95% CI: 20.4%-34.1%) compared with those who did not at 4.9% (15 of 305, 95% CI: 2.8%-8.0%; *P* < 0.001). This was primarily driven by a higher TLR rate among patients undergoing surveillance angiography 21.7% (38 of 175, 95% CI: 15.8%-28.6%) vs those who did not at 3.3% (10 of 305, 95% CI: 1.6%-5.9%) (Figure 3, Table 7). The Kaplan-Meier estimate of cumulative MACE at 1 year for patients who did not undergo surveillance angiography is shown in Figure 6. Stratified by lesion type, MACE occurred 5.8% (12 of 206, 95% CI: 3.0%-10.0%) of patients with ISR and 3.1% (3 of 97, 95% CI: 0.6%-8.8%) of patients with de novo lesions. In patients undergoing protocol-mandated surveillance angiography who subsequently underwent TLR, core laboratory QCA at protocol-mandated 9-month follow-up demonstrated in-lesion diameter stenosis <50% in 4.1% (2 of 49, 95% CI: 0.5%-14.0%), 50% to 70% in 77.6% (38 of 49, 95% CI: 63.4%-88.2%), and >70% in 18.4% (9 of 49, 95% CI: 8.8%-32.0%) (Table 8).

**Table 7:** 12-Month Clinical Outcomes With and Without Surveillance Angiography

	9-Month Surveillance Angiography Performed					
	Yes			No		
	Overall N = 175	ISR n = 81	De Novo n = 92	Overall N = 305	ISR n = 206	De Novo n = 97
MACE	47 (26.9) [20.4-34.1]	31 (38.3) [27.7-49.7]	16 (17.4) [10.3-26.7]	15 (4.9) [2.8-8.0]	12 (5.8) [3.0-10.0]	3 (3.1) [0.6-8.8]
Cardiac death	0 (0.0) [0.0-2.1]	0 (0.0) [0.0-4.5]	0 (0.0) [0.0-3.9]	2 (0.7) [0.1-2.3]	2 (1.0) [0.1-3.5]	0 (0.0) [0.0-3.7]
Myocardial infarction	3 (1.7) [0.4-4.9]	3 (3.7) [0.8-10.4]	0 (0.0) [0.0-3.9]	5 (1.6) [0.5-3.8]	3 (1.5) [0.3-4.2]	2 (2.1) [0.3-7.3]
TVR	44 (25.1) [18.9-32.2]	28 (34.6) [24.3-46.0]	16 (17.4) [10.3-26.7]	11 (3.6) [1.8-6.4]	9 (4.4) [2.0-8.1]	2 (2.1) [0.3-7.3]
TLR	38 (21.7) [15.8-28.6]	23 (28.4) [18.9-39.5]	15 (16.3) [9.4-25.5]	10 (3.3) [1.6-5.9]	9 (4.4) [2.0-8.1]	1 (1.0) [0.0-5.6]

Values are reported as n (%); 95% CIs for binary clinical outcomes were calculated using the Clopper-Pearson exact method, were not adjusted for multiplicity, and are reported as [lower confidence interval, upper confidence interval].

TLR = target lesion revascularization; TVR = target vessel revascularization; other abbreviations as in Tables 1 and 6.

**Table 8:** 12-Month Target Lesion Revascularization Events Stratified by Restenosis Severity at 9-Month Follow-Up Quantitative Coronary Angiography

Characteristics	Overall N = 49	ISR n = 31	De Novo n = 18
In-lesion diameter stenosis: <50%	2 (4.1) [0.5-14.0]	1 (3.2) [0.1-16.7]	1 (5.6) [0.1-27.3]
In-lesion diameter stenosis: 50%-70%	38 (77.6) [63.4-88.2]	26 (83.9) [66.3-94.5]	12 (66.7) [41.0-86.7]
In-lesion diameter stenosis: >70%	9 (18.4) [8.8-32.0]	4 (12.9) [3.6-29.8]	5 (27.8) [9.7-53.5]

Values are reported as n (%) unless otherwise noted; 95% CIs for binary clinical outcomes were calculated using the Clopper-Pearson exact method, were not adjusted for multiplicity, and are reported as [lower confidence interval, upper confidence interval].

Abbreviation as in Table 1.

## Discussion

The findings of this study suggest the AGENT DCB is safe and effective in ethnically Chinese patients, with a 12-month MACE rate of 13.9%, consistent with expectations for real-world registries. Beyond the overall MACE rate, these results highlight 2 clinically relevant themes: the higher inherent risk of ISR relative to de novo disease and the extent to which follow-up strategy can shape revascularization-driven endpoints (Central Illustration). A trend toward a higher MACE rate in ISR compared with de novo patients was observed, reflecting the higher-risk profile of these lesions, consistent with prior studies.^8^ De novo patients with multivessel disease and patients with diabetes demonstrated higher 12-month event rates compared with those without, indicative of their higher clinical risk.

**Central Illustration:**
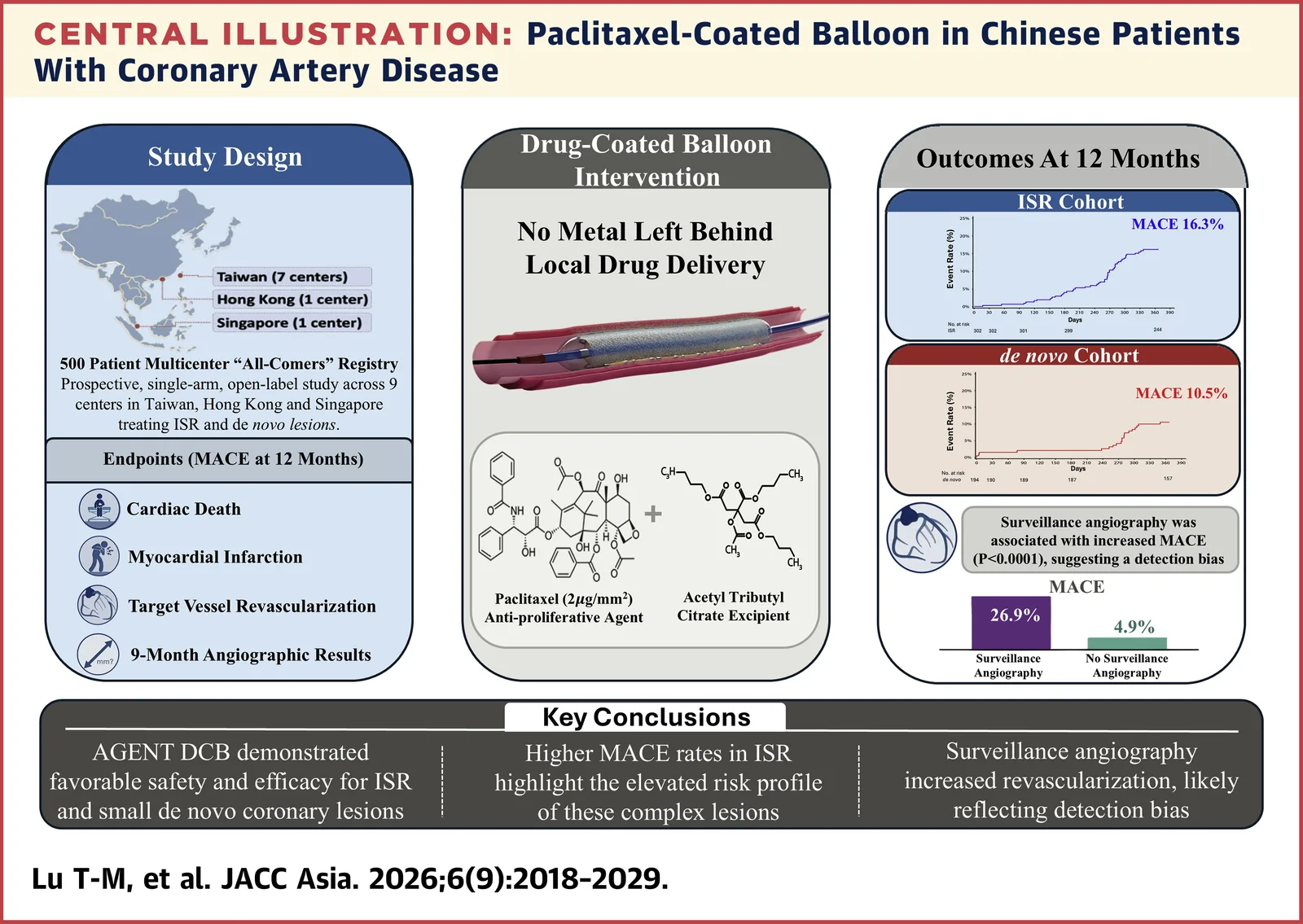
Paclitaxel-Coated Balloon in Chinese Patients With Coronary Artery Disease In this prospective, multicenter registry of 500 ethnically Chinese patients with in-stent restenosis (ISR) or de novo coronary artery disease treated with a paclitaxel-coated balloon, 12-month major adverse cardiac events (MACEs) occurred in 13.9% overall, including 16.3% in ISR and 10.5% in de novo patients. Cardiac death, myocardial infarction, and target vessel revascularization occurred in 1.6%, 2.0%, and 11.7%, respectively. Surveillance angiography was associated with substantially higher 12-month MACE as compared with not receiving surveillance angiography (26.9% vs 4.9%), primarily driven by higher target lesion revascularization rates (21.7% vs 3.3%), underscoring the influence of follow-up imaging on revascularization-driven outcomes. Overall, paclitaxel-coated balloon treatment appeared safe and effective in this real-world Chinese population. DCB = drug-coated balloon.

There was a higher rate of revascularization events observed in patients who underwent surveillance angiography compared with those who did not. This likely reflects detection bias, a well-recognized phenomenon in PCI studies. This bias may be especially pronounced in the context of DCB angioplasty, where decisions regarding re-intervention are strongly influenced by visual cues from angiography.^11^ Previous randomized studies have reported similar binary angiographic restenosis rates between DCB and DES, yet re-intervention was more common in the DCB arms.^12^ This may reflect a clinical reluctance to implant additional metallic layers in restenotic lesions, even in the presence of angiographic restenosis, and demonstrates how DCB may preserve future treatment options.^12^ An important interpretive consideration is that TLR in this registry was not restricted to “clinically driven” events; therefore, repeat revascularization could be triggered by restenosis identified on protocol-mandated surveillance angiography, even in the absence of recurrent symptoms or objective ischemia. Contemporary Academic Research Consortium DCB endpoint guidance emphasizes separating angiographic findings from clinical events by defining TLR preferentially as clinically driven (with physiologic confirmation when feasible), to reduce ascertainment bias introduced by routine angiographic follow-up.^13^ In this context, a severity-stratified analysis was performed to describe the degree of angiographic restenosis at 9-month follow-up among surveillance angiography patients who underwent TLR by 12 months. The key observation is that TLR in the surveillance patients was predominantly associated with intermediate-range restenosis, with a smaller proportion associated with high-grade or mild restenosis. This distribution is clinically relevant because contemporary endpoint guidance generally expects physiologic corroboration to support revascularization decisions when stenosis is intermediate, particularly when angiography is performed without antecedent ischemia testing as may occur with protocol-mandated follow-up.^13^ Collectively, these findings suggest that routine surveillance angiography may have preferentially increased detection and shifted the treatment threshold toward subsequent intervention of intermediate restenosis in this study, contributing to higher observed revascularization-driven event rates compared with a strategy that relies primarily on clinical presentation and objective ischemia. These findings raise important questions about how surveillance imaging influences clinical outcomes and decision thresholds in DCB-treated patients.

In the de novo cohort, 9-month core-lab QCA showed modest average late lumen loss. Although 37% of lesions experienced LLE, the mean late lumen loss contrasts with some reports of mean late lumen gain after paclitaxel-coated balloon in de novo disease.^14^ In the AGENT Japan small vessel randomized trial, de novo patients treated with the study device had less severe baseline diameter stenosis (68.2% ± 11.0%), less diabetes (35.6%), achieved a strong acute result with low residual stenosis (23.0% ± 10.5%), and demonstrated favorable mean 6-month in-lesion late loss (−0.03 ± 0.34 mm).^14^ In contrast, de novo patients in the present registry appear to have a more severe baseline substrate (diameter stenosis 85.7% ± 10.1%), more diabetes (50.5%), and an acute result of 31.9% ± 12.2% residual diameter stenosis. Taken together, these differences in baseline complexity and acute procedural result provide a plausible explanation for the differences in biologic vessel response following paclitaxel-coated balloon treatment. Nevertheless, despite the lower LLE rate in our study, LLE remained numerically higher than that reported with a sirolimus-coated balloon (∼30%) in the randomized TRANSFORM I (TReAtmeNt of Small coronary vessels: Randomized controlled trial FOR MagicTouch sirolimus coated balloon) trial.^15^

Overall, these real-world clinical outcomes are broadly consistent with prior coronary DCB experiences reported in randomized trials, although between-study comparisons should be interpreted cautiously. Among DCB-treated ISR patients in the AGENT IDE, the 1-year target lesion failure rate was 17.9%, and in the DAEDALUS (Difference in Anti-restenotic Effectiveness of Drug-eluting stent and drug-coated balloon AngiopLasty for the occUrrence of coronary in-Stent restenosis) meta-analysis the 1-year target lesion revascularization rate was 11.1%.^2^^,^^12^ In REC-CAGEFREE I (Drug-Coated Balloon Angioplasty With Rescue Stenting Versus Intended Stenting for the Treatment of Patients With De Novo Coronary Artery Lesions), the 1-year event rate was 3.6% among small vessel de novo DCB patients for the composite endpoint of cardiovascular death, target vessel MI, and clinically and physiologically indicated target lesion revascularization.^7^ However, there was no protocol-mandated surveillance angiography.^7^ In the present study, the 1-year MACE rate in de novo patients was 10.5% overall, but only 3.2% among those without surveillance angiography. Collectively, the outcomes observed in this registry are aligned with the broader DCB literature, including previous studies in ethnically Chinese patients, and emphasize that endpoint definitions and protocol-mandated follow-up can meaningfully shape reported event rates.^7^

Although this was an all-comers trial, most de novo lesions enrolled were for small vessel disease. In subgroup analysis, de novo small vessels are stratified by ≤2.75 mm for consistency with historical and upcoming clinical trials for the study device and account for 184 de novo patients (95%).^6^^,^^8^^,^^9^ More clinical data in other de novo lesion types is warranted to further validate the clinical effectiveness of paclitaxel-coated balloon for all-comers. The ongoing global AGENT STANCE IDE trial (AGENT Drug-Coated Balloon for STent AvoidANCE in PCI for De Novo Coronary Artery Disease; NCT06959524), a randomized comparison of paclitaxel-coated balloon vs DES in de novo lesions, will be critical to validate and expand on these findings. In this article, we report results of a multicenter registry that complement the ongoing STANCE IDE randomized trial by providing real-world evidence from routine clinical practice, encompassing a broad range of lesion complexity in both de novo and ISR patients and reflecting treatment decision-making outside the constraints of a randomized controlled trial. The STANCE IDE will include diverse lesion subsets such as small vessels (≤2.75 mm), bifurcations, and long diffuse lesions, helping to further define the role of paclitaxel-coated balloon across a broad de novo clinical spectrum.

### Study limitations

First, this was a single-arm observational study without a comparator group, which limits the ability to assess comparative effectiveness. Second, a notable disparity in MACE rates was observed between patients who underwent routine surveillance angiography and those who did not, likely reflecting detection bias introduced by routine, protocol-mandated follow-up imaging. Repeat revascularization endpoints were not restricted to clinically driven events in this study. Recent studies have addressed this issue by defining TLR as “clinically or physiologically” indicated rather than including all TLR events.^7^ Because symptom status, objective ischemia testing, and/or physiologic lesion assessment were not uniformly captured at the time of repeat angiography or revascularization, we cannot definitively distinguish angiography-driven from clinically driven TLR on an event-by-event basis. Consistent with ARC recommendations, future studies in DCB-treated populations may benefit from prospectively adjudicating repeat revascularization using clinically and/or physiologically driven criteria, particularly when protocol-mandated angiographic follow-up is incorporated.^13^ Third, the relatively high residual stenosis observed after DCB treatment in some lesions, particularly in de novo disease, may reflect lesion complexity and variability in lesion preparation. The study did not systematically incorporate a standardized lesion preparation strategy, which may influence acute procedural results and subsequent outcomes. Finally, the study did not incorporate the results of intravascular imaging, which has been shown to improve both procedural and long-term outcomes, as well as follow-up angiographic results in DCB PCI.^16^^,^^17^ Despite these limitations, this study represents the first real-world registry evaluating the study device paclitaxel-coated balloon in an ethnically Chinese all-comer population, providing valuable insights into its performance in contemporary clinical practice.

## Conclusions

The use of paclitaxel-coated balloon for all-comers PCI in an ethnically Chinese population was safe and effective in this large, real-world registry. Clinical outcomes were consistent across key subgroups including male vs female patients, ISR vs de novo, as well as those with acute coronary syndrome or long lesions (>25 mm). There was a trend toward higher 12-month MACE in ISR patients, and de novo patients with multivessel disease or diabetes were also at higher risk, reflecting their overall clinical complexity. These findings support the broader application of paclitaxel-coated balloon in diverse real-world settings and provide timely data to inform clinical practice in East Asian populations—which is particularly important in light of the study device's recent regulatory approval in China.

## Funding Support and Author Disclosures

This study was funded by Boston Scientific Corporation. Dr Rafael Cavalcante and Jack Livingston are full-time employees with equity interest in Boston Scientific Corporation. All other authors have reported that they have no relationships relevant to the contents of this paper to disclose.

## References

[1] Madhavan M.V., Kirtane A.J., Redfors B. (2020). Stent related adverse events 1 year after percutaneous coronary intervention. J Am Coll Cardiol.

[2] Yeh R.W., Shlofmitz R., Moses J. (2024). Paclitaxel-coated balloon vs uncoated balloon for coronary in-stent restenosis: the AGENT IDE randomized clinical trial. JAMA.

[3] Giacoppo D., Alfonso F., Xu B. (2020). Paclitaxel-coated balloon angioplasty vs. drug-eluting stenting for the treatment of coronary in-stent restenosis: a comprehensive, collaborative, individual patient data meta-analysis of 10 randomized clinical trials (DAEDALUS study). Eur Heart J.

[4] Fezzi S., Giacoppo D., Fahrni G. (2025). Individual patient data meta-analysis of paclitaxel-coated balloons vs. drug-eluting stents for small-vessel coronary artery disease: the ANDROMEDA study. Eur Heart J.

[5] Gao X., Tian N., Kan J. (2025). Drug-coated balloon angioplasty of the side branch during provisional stenting: the multicenter randomized DCB-BIF trial. J Am Coll Cardiol.

[6] Nakamura M., Isawa T., Nakamura S. (2024). One-year safety and effectiveness of the agent paclitaxel-coated balloon for the treatment of small vessel disease and in-stent restenosis. Cardiovasc Interv Ther.

[7] Gao C., He X., Ouyang F. (2024). Drug-coated balloon angioplasty with rescue stenting versus intended stenting for the treatment of patients with de novo coronary artery lesions (REC-CAGEFREE I): an open-label, randomised, non-inferiority trial. The Lancet.

[8] Iannopollo G., Giannini F., Ponicelli F. (2020). Percutaneous coronary intervention with the agent paclitaxel-coated balloon: a real-world multicenter experience. J Invasive Cardiol.

[9] Tervo J., Kärkkäinen J.M., Rissanen T.T. (2022). Technical success, clinical efficacy, and insight into the causes of restenosis after the percutaneous coronary intervention of de novo coronary artery lesions using a paclitaxel-coated balloon with citrate ester excipient. Front Cardiovasc Med.

[10] Cutlip D.E., Windecker S., Mehran R. (2007). Clinical end points in coronary stent trials: a case for standardized definitions. Circulation.

[11] Misumida N., Kobayashi A., Kim S.M., Abdel-Latif A., Ziada K.M. (2017). Role of routine Follow-up coronary angiography after percutaneous coronary intervention—systematic review and meta-analysis. Circ J.

[12] Kereiakes D.J., Kandzari D.E., Zidar J.P. (2020). Drug-coated balloons for in-stent restenosis. J Am Coll Cardiol.

[13] Fezzi S., Scheller B., Cortese B. (2025). Definitions and standardized endpoints for the use of drug-coated balloon in coronary artery disease: consensus document of the drug coated balloon academic research consortium. Eur Heart J.

[14] Nakamura M., Isawa T., Nakamura S. (2023). Drug-coated balloon for the treatment of small vessel coronary artery disease—a randomized non-inferiority trial. Circ J.

[15] Ninomiya K., Serruys P.W., Colombo A. (2023). A prospective randomized trial comparing sirolimus-coated balloon with paclitaxel-coated balloon in de novo small vessels. Cardiovasc Interv.

[16] Lee J.M., Choi K.H., Song Y.B. (2023). Intravascular imaging–guided or angiography-guided complex PCI. N Engl J Med.

[17] Gao X.F., Ge Z., Kong X.Q. (2024). Intravascular ultrasound vs angiography-guided drug-coated balloon angioplasty: the ULTIMATE Ⅲ trial. Cardiovasc Interv.

