# Supplementary material for: Paclitaxel-Coated Balloon for Coronary In-Stent Restenosis and De Novo Disease in Chinese Patients
Source: JACC Asia. 2026 Aug 1;6(9):2018–29. doi: 10.1016/j.jacasi.2026.06.008 (PMC13554778; doi:10.1016/j.jacasi.2026.06.008)
Supplement: Supplementary file 1 [file mmc1.docx]

**Supplemental Table S1. Members of Clinical Events Committee**

| Member | Affiliation |
| --- | --- |
| Weifeng Shen, MD | Shanghai Ruijin Hospital, Shanghai, China |
| Jie Qian, MD | Fuwai Hospital Chinese Academy of Medical Sciences, Beijing, China |
| Kefei Dou, MD | Fuwai Hospital Chinese Academy of Medical Sciences, Beijing, China |
| Shubin Qiao, MD | Fuwai Hospital Chinese Academy of Medical Sciences, Beijing, China |
| Xing Biao Qiu, MD | Shanghai Chest Hospital, Shanghai, China |

**Supplemental Figure S1:** Patient Disposition through one year. A total of 500 “all-comer” consecutive patients were enrolled. 489 patients (97.8%) had clinical follow-up or death; among those without 1-year follow-up, 1 was lost to follow-up, 5 withdrew participation, 2 missed the follow-up visit with no later follow-up performed, 2 missed 1-year follow-up with later follow-up visit performed, and 1 patient did not use AGENT DCB. 9-month surveillance angiography was performed in 92 (44.5%) de novo patients and 81 (27.2%) ISR patients.
